# Diagnosis of *Helicobacter pylori* Infection in a Routine Testing Workflow: Effect of Bacterial Load and Virulence Factors

**DOI:** 10.3390/jcm10132755

**Published:** 2021-06-23

**Authors:** Nabil Gastli, Margaux Allain, Dominique Lamarque, Vered Abitbol, Annick Billoët, Gislène Collobert, Romain Coriat, Benoit Terris, Nicolas Kalach, Josette Raymond

**Affiliations:** 1 Service de Bactériologie, AP-HP Centre-Université de Paris, Hôpital Cochin, 75014 Paris, France; nabil.gastli@aphp.fr (N.G.); marguax.allain@gmail.com (M.A.); annick.billoet@aphp.fr (A.B.); gislene.collobert@aphp.fr (G.C.); 2 Gastroentérologie, Hôpital Ambroise Paré, AP-HP, Université Versailles-Saint Quentin-en-Yvelines, 92100 Boulogne, France; dominique.lamarque@aphp.fr; 3 Gastroentérologie, AP-HP Centre-Université de Paris, Hôpital Cochin, 75014 Paris, France; vered.abitbol@aphp.fr (V.A.); romain.coriat@aphp.fr (R.C.); 4 Anatomopathologie, AP-HP Centre-Université de Paris, Hôpital Cochin, 75014 Paris, France; benoit.terris@aphp.fr; 5 Clinique pédiatrique Saint Antoine, Hôpital Saint Vincent de Paul, Groupement des Hôpitaux de l’Institut Catholique de Lille (GHICL), 59020 Lille, France; kalach.nicolas@ghicl.net; 6 Service de Bactériologie, AP-HP Université Paris-Saclay, Hôpital Bicêtre, 94270 Le Kremlin-Bicêtre, France; 7 Hôpital de Bicêtre, 78 Rue du Général Leclerc, 94270 Le Kremlin-Bicêtre, France

**Keywords:** *Helicobacter pylori*, *cagA* gene, PCR, diagnosis, histology, culture

## Abstract

Reliable diagnostic methods are mandatory for effective management of *Helicobacter *pylori** infection. Histology and culture are the most common invasive methods in current practice, even if molecular methods are gaining in importance. The performance of these conventional methods varies significantly. We conducted a retrospective study of 1540 adults and 504 children with gastric biopsies taken during endoscopy to assess the impact of bacterial load and the *cagA* virulence factor on the performance of *H. pylori* infection testing. The association between virulence and histology findings was also investigated. With 23S rRNA qPCR confirmed by *glmM* amplification as the gold standard, culture and histology had lower sensitivity, 74.4% and 73.3%, respectively. However, their sensitivity was enhanced (>90%) in biopsies with high bacterial load (qPCR Ct < 30). Positive *cagA* status of the strain was associated with high bacterial load (94.9%), thus resulting in more frequent positive culture (94.3%) and *H. pylori* histology detection (91.7%) and more severe lesions on histology (*p* < 0.001). Conversely, the *cagA* status of the strains was negative in 110/119 (92.4%) of biopsies with low bacterial load (qPCR Ct < 30), 82/90 (91.1%) with negative *H. pylori* histology detection and 119/131 (90%) with negative culture findings (*p* < 0.001). This study highlights the low sensitivity of conventional culture and histology that may lead to false negative diagnosis if used alone. *H. pylori* quantification associated with *cagA* genotyping in routine workflow are essential for a sensitive and reliable diagnosis, to identify patients at high risk and to manage eradication therapies.

## 1. Introduction

*Helicobacter pylori* infection is related to a wide spectrum of diseases including uncomplicated or complicated ulcer diseases, mucosa-associated lymphoid tissue lymphoma, atrophic gastritis and gastric cancer. The correct management of these severe conditions requires constant collaboration between gastroenterologists, pathologists and biologists.

*H. pylori* infection can be diagnosed by the non-invasive techniques serology, 13C urea breath test and stool antigen test, even if the strategy “test and treat” is no longer recommended. The other approach requires gastric biopsy collection during endoscopy and then analysis by urease tests, histopathology, culture and/or molecular detection by PCR [1]. Histology allows for detecting *H. pylori* and tissue damage and is one of the most commonly used methods. Indeed, the Maastricht consensus states that most cases can be diagnosed by only histochemical staining of biopsies [2].

*H. pylori* culture is essential for in vitro susceptibility testing but requires well-trained laboratory staff. Molecular methods allow for high sensitivity and detection of both *H. pylori* DNA and specific point mutations associated with clarithromycin resistance. This promotes easier administration of a tailor-made treatment as recommended [2]. This approach also offers the possibility of quantifying bacterial load and screening virulence factors.

In 2011, Espinoza et al. recommended the use of the *glmM* gene for detecting *H. pylori* [3]. Indeed, the housekeeping gene *glmM* is essential for bacterial growth and cell-wall synthesis and is therefore specific to *H. pylori* [4]. Hence, we systematically used *H. pylori*-specific *glmM* PCR to confirm qPCR results.

Among multiple virulence factors identified in *H. pylori*, two main factors associated with gastric epithelial cell pathogenicity have been extensively studied: cytotoxin-associated gene A (CagA) protein, coded by the *cagA* gene that is part of the cag pathogenicity island (cag-PAI), and the pore-forming cytotoxin called vacuolating cytotoxin (VacA) [5]. CagA is directly injected into epithelial cells via a type IV secretion system and undergoes tyrosine phosphorylation within repeated sequences of five amino acids (glutamic acid, proline, isoleucine, tyrosine, alanine), called EPIYA motifs. The phosphorylated form is able to deregulate normal cellular signaling. The *cagA* gene is grouped into two different allele types: Western alleles that encode one or multiple EPIYA-C motifs and East Asian alleles that encode one EPIYA-D motif [6].

The severity of gastric lesions was suggested to be associated with *cagA* status and *vacA* polymorphism [7,8,9]. Moreover, quantitative molecular methods in earlier observations showed an association between *cagA* status and bacterial density, thus suggesting a potential impact of virulence factors on performance of the diagnostic method [10].

The aim of our study was to assess, in a large French cohort of adults and children, the genotypes of *cagA* and *vacA* and investigate their association with histology findings and their effect on the performance of conventional methods for bacterial detection.

## 2. Material and Methods

### 2.1. Patients, Endoscopy and Gastric Biopsy Sampling

From January 2017 to April 2019, 2044 patients (1540 adults and 504 children), all presenting gastrointestinal disorders, underwent upper gastrointestinal endoscopy, with gastric biopsy sampling and testing for *H. pylori* infection performed in the bacteriology laboratory of Cochin Hospital (Paris, France). Adults attended the gastroenterology units of Cochin Hospital or Ambroise Paré Hospital (Boulogne, France), whereas children attended the Saint Vincent de Paul Hospital (Lille, France). Biopsies were taken from both the gastric antrum (*n =* 4) and corpus (*n =* 4). Patients who had received antibiotics during the previous 4 weeks or proton pump inhibitors (PPIs) during the previous 2 weeks were excluded. All samples were submitted for routine diagnostics. Patients were investigated in a hospital setting, according to good clinical practices.

Written informed consent for the endoscopic and diagnostic procedures was obtained from patients or their parents/legal guardians and kept in their medical records. The study was carried out in care structures, which have a CNIL correspondent who ensures data compliance. No extra biopsy sampling or additional endoscopies were required, and all samples were anonymized. This is a retrospective study therefore non-interventional study. Furthermore, in France no declaration to the CNIL is needed if more than 1000 patients are included.

### 2.2. Culture

One antral and one corpus biopsy specimen were taken for bacteriological analysis. The two biopsies were homogenized in a glass Griffiths tube in 0.5 mL of brain heart infusion medium (bioMérieux, Marcy l’Etoile, France). One part of the suspension was plated on Schaedler agar with vitamin K1 and 5% sheep’s blood (Becton Dickinson, Heidelberg, Germany) and the PYL selective medium (bioMérieux, Marcy l’Etoile, France). After inoculation, plates were immediately incubated at 37 °C for 10 days under wet and microaerophilic conditions.

The other part of the suspension was kept for molecular methods.

*H. pylori* growth was monitored starting from day 3. Positive cultures were confirmed as *H. pylori* on the basis of Gram staining, colony morphology (small, round colonies) and positive oxidase, catalase and urease reactions. Strains were stored at −80 °C in 3 mL meat-liver media (bioMérieux, Marcy l’Etoile, France) with 0.3 mL glycerol added.

### 2.3. Histology

Three antral and three corpus specimen were formalin-fixed and paraffin-embedded. Tissue sections were stained with Giemsa for detection of *Helicobacter*-like organisms and hematoxylin-eosin (H&E) for histological grading. Immunohistochemical staining was performed only in cases of severe gastritis or suspected mucosa-associated lymphoid tissue lymphoma or gastric cancer. An experienced expert pathologist read the slides according to the routine pathology laboratory workflow. Therefore, slides were not reviewed by another pathologist in case of discordant results with culture/molecular methods. The degree of inflammation (mononuclear cell infiltration), activity (neutrophil infiltration), gastric glandular atrophy and intestinal metaplasia was assessed according to the updated Sydney classification as follows: 0, absent; 1, mild: 2, moderate; 3, marked [11]. Presence of erosions or lymphoid follicles was noted. Then, patients were classified into 3 categories: “normal histology” (grade 0), “mild and moderate gastritis” (grade 1–2), “severe gastritis and gastric cancer” (grade 3).

### 2.4. Detection of H. pylori by qPCR

An in-house qPCR assay was used to detect both the presence of *H. pylori* and point mutations conferring clarithromycin resistance as described [12]. From the homogenized suspension, DNA was isolated by using a QIAamp DNA mini kit (Qiagen SA, Courtaboeuf, France). A 267-bp fragment of the *H. pylori* 23S rRNA gene was amplified with primers for HPYS and HPYA (Table 1) using the LightCycler thermocycler (Roche Diagnostics, Neuilly sur Seine, France). The specificity of the qPCR method was evaluated by using different microorganisms (*Enterococcus faecalis, Pseudomonas aeruginosa*, coagulase-negative *Staphylococcus*, *Candida albicans*, *Escherichia coli, Klebsiella pneumoniae)* for which qPCR testing remained negative. The qPCR testing was also negative for *Campylobacter* spp. Each run included positive and negative controls, the former prepared from 10^−2^, 10^−4^, and 10^−6^ dilutions of 45 μg/mL DNA from *H. pylori* strain H37Rv, and the latter consisting of sterile water. Quality control was acceptable when the negative control had an undetectable cycle threshold (Ct) and the 10^−2^, 10^−4^, and 10^−6^ dilutions of *H. pylori* DNA McFarland 1 had Ct values of 17–19, 19–27, and 27–33, respectively [13].

### 2.5. Confirmation of H. pylori Detection by Amplifying glmM

*H. pylori*-specific *glmM* PCR was systematically performed to confirm qPCR results [14]. A 294-bp fragment internal to *glmM* was amplified as described (Table 1). Molecular techniques (23S rRNA confirmed by *glmM*) were considered the gold standard in this study for diagnosis of *H. pylori* infection [15].

### 2.6. cagA Status

The *cagA* status was determined on all DNA extracts by a specific PCR with a set of primers for *cagF* and *cagR* (Table 1). A 349-bp fragment from the middle conservative region of the *cagA* gene was amplified in *cagA*-positive strains [16]. An empty-site assay with primers 2 and 25 (Table 1), which flank the left and right ends of the cag-PAI, was used to confirm the absence of the pathogenicity island cag-PAI in *cagA*-negative strains [17]. PCR products for each reaction were analyzed by gel electrophoresis.

### 2.7. Characterization of the C-Terminal Variable Region by EPIYA Motifs

To assess the number and type of EPIYA motifs in *cagA*-positive samples, separate PCR reactions involved using a common forward primer for *cagA*28F and the reverse primers for cagA-P1C, cagA-P2TA and cagA-P2CG (1:1 mixture) and cagA-P3E (Table 1) for amplifying EPIYA-A, -B, -C and -D, respectively [18]. To distinguish between EPIYA-C and -D, another reaction with primers for *cagA*28F and cagA-pD (Table 1) was performed to identify the presence of an EPIYA-D motif [19]. Resulting patterns were visualized by ethidium bromide staining after electrophoresis on a 1.5% agarose gel. In case of inconclusive PCR genotyping and for some randomly selected *cagA*-positive samples, the 3′ *cagA* variable region was amplified, then sequenced with the primers for cagA2530S and cagA3000AS (Table 1) as described [20]. All in-house PCR amplicon sequencing was performed by a custom sequencing service (Eurofins MWG Operon, Ebersberg, Germany).

### 2.8. vacA Genotyping

Polymorphisms in the signal (s-) and middle (m-) region of *vacA* were determined as described [16]. Typing of the *vacA* s- sequence region was characterized by using a set of primers for VA1F and VA1R (Table 1), which resulted in generating fragments of 259 bp for type s1 variants or fragments of 286 bp for type s2 variants. The *vacA* m- region was typed by using a set of primers for VAG-F and VAG-R to amplify a 570-bp product for m1 and 645-bp product for m2 (Table 1). PCR products for each reaction were analyzed by gel electrophoresis.

### 2.9. Statistical Analysis

The online software VassarStats (Richard Lowry) was used for data analysis. Frequencies of categorical variables were calculated, and Fisher’s exact test, chi-square test or Student *t* test was used to determine differences between groups. Statistical significance was set at *p* < 0.05.

## 3. Results

### 3.1. Patients and H. pylori Infection

During the study period, 1540 adults (744 men and 796 women; mean age 52.6 ± 16.5 years (16 years–94 years)) and 504 children (261 boys and 243 girls, mean age 8.4 ± 4.4 years (8 months–17 years)) underwent endoscopy.

*H. pylori* culture was positive in 382 biopsies: 346/1540 (22.4%) from adults and 36/504 (7.1%) from children. The molecular techniques (in-house qPCR confirmed by the *glmM* PCR) proved infection in 513 biopsies: 453/1540 (29.4%) from adults and 60/504 (11.9%) from children. Therefore, *H. pylori* infection was proved only by molecular methods in 107 adults and 24 children.

*H. pylori* positivity rates were significantly higher in adults than children (*p* < 0.001), but rates did not differ by sex or age within each group. With the in-house qPCR confirmed by the *glmM* PCR as the reference, culture showed a sensitivity and specificity of 74.4% and 100%, respectively. When considering only high-bacterial-load biopsies (i.e., qPCR Ct < 30), the sensitivity of culture increased to 93.4%. Most biopsies (105/131, 80.1%) with a false negative culture had low bacterial load (i.e., qPCR Ct ≥ 30) (Table 2).

### 3.2. Histology

Histology reports were available for only 338 *H. pylori*-positive patients: 278 adults attending the gastroenterology unit of Cochin Hospital and 60 children attending the Saint Vincent de Paul Hospital in Lille. As compared with molecular techniques, *Helicobacter*-like organisms histology detection had a sensitivity of 73.3% (248/338); the specificity could not be calculated because histology data for only *H. pylori*-positive patients were collected. Logically, direct examination with Giemsa staining was more sensitive (234/253, 92.5%) in biopsies with high bacterial load (qPCR Ct < 30), whereas a low bacterial load (qPCR Ct ≥ 30) was associated with absence of *Helicobacter*-like organisms on histology (71/85, 83.5%) (Table 2).

The distribution of adults and children infected by *Helicobacter*-like organisms by histology grading is shown in Table 3. Among the 60 children, 18 (30%) had normal findings, 28 (46.6%) mild or moderate gastritis and 14 (23.3%) severe gastritis. In the same way, among the 278 adults, 23 (8.2%) had normal findings, 204 (73.3%) mild or moderate gastritis and 51 (18.3%) severe gastritis including 2 with gastric cancers. For both groups, the presence of *Helicobacter*-like organisms on histology sections was significantly associated with gastritis severity: 3/41 (7.3%) with normal findings vs. 191/232 (82.3%) and 54/65 (83%) with moderate and severe gastritis, respectively (*p* < 0.01) (Table 3). Of note, high bacterial load (qPCR Ct < 30) was significantly associated with gastritis severity: 5/41 (12.2%) with normal findings vs. 193/232 (83.2%) and 56/65 (86.1%) with moderate and severe gastritis, respectively (*p* < 0.01) (Table 3).

### 3.3. cagA Status and Genotype

The *cagA* status was successfully assessed in 495/513 *H. pylori*-positive biopsies (435/453 from adults and 60/60 from children) and was undetermined for 18 adults. Among these 495 biopsies, 158 (31.9%) were *cagA*-positive (151 from adults and 7 from children). Therefore, the prevalence of the *cagA* gene was significantly lower in children than adults (11.6% vs. 34.7%; *p* < 0.001). The absence of cag-PAI was confirmed by empty-site PCR in all *cagA*-negative samples. Among *cagA*-positive isolates, all EPIYA motif patterns were related to the Western type, harboring the ABC (*n* = 122, 80.8%), ABCC (*n* = 12), BC (*n* = 6), AC (*n* = 6), ABC+ABCC (*n* = 2), AB (*n* = 1), ACC (*n* = 1) and BCC (*n* = 1) motifs in adults and ABC (*n* = 6, 85.7%) and AC (*n* = 1) motifs in children. For both groups, *cagA* status was significantly associated with gastritis severity: 3/41 (7.3%) with normal findings vs. 63/232 (27.1%) and 30/65 (46.1%) with moderate and severe gastritis, respectively (*p* < 0.01) (Table 3).

### 3.4. vacA Genotype and Association with cagA Status

Data on *vacA* genotyping were available for only 292 and 30 *H. pylori*-positive biopsies from adults and children, respectively. The most frequent allele combination was *s1m1* (51.2%, 165/322), followed by *s2m2* (31.7%, 102/322), *s1m2* (12.1%, 39/322) and *s2m1* (5.0%, 16/322). The distribution of *vacA* allelic variants was homogeneous among adults and children, and no mixed infection with different variants was found. We found a significant association between *vacA* allele combinations and *cagA* status: 61.8% (102/163) of *s1m1* variants were *cagA*-positive and 93.1% (95/102) of *s2m2* variants were *cagA*-negative (*p* < 0.001).

### 3.5. Association between Presence of H. pylori and Histology, Bacterial Load (qPCR), cagA Status of the Strain and Severity of Histology Lesions

We observed a significant association between the severity of histology lesions and both the presence of *Helicobacter*-like organisms on histology and bacterial load expressed by qPCR Ct values (7.3% to 83% and 12.2% to 86.1%, respectively, *p* < 0.001, for normal histology findings to severe gastritis) (Table 3). The prevalence of *cagA* gene increased significantly from 7.3% (3/41) with normal histology findings to 46.1% (30/65) with severe gastritis/gastric cancer (Table 3). Of note, one patient in two with gastric cancer carried a *cagA*-negative strain and all of the children with normal histology findings carried a *cagA*-negative strain.

### 3.6. Association between a Positive Culture, Bacterial Load (qPCR), Presence of H. pylori on Histology and cagA Status of the Strain

We found a significant association between the presence of *Helicobacter*-like organisms on histology, a positive culture and the *cagA* status of the strain (all *p* < 0.001) (Table 4). Indeed, as compared with *cagA*-negative isolates, *cagA*-positive strains were more often positive on histology (91.7% vs. 64.5%) and culture (94.3% vs. 64.7%) and had high bacterial load (94.9% vs. 67.3%). Notably, 76/84 (90.5%) biopsies *H. pylori*-positive by PCR and negative on histology had a *cagA*-negative status. In the same way, 119/128 (92.9%) biopsies *H. pylori*-positive by PCR and negative culture had a *cagA*-negative status. In summary, *H. pylori* bacterial load, expressed by qPCR Ct values, was significantly associated with the *cagA* status of strains (150/158, 94.9%), and biopsies with high bacterial load (qPCR Ct < 30) were *cagA*-positive, (*p* < 0.001). (Table 4). Conversely, in 119 biopsies with low bacterial load (qPCR Ct ≥ 30), only eight (6.7%) were positive for *cagA*. Overall, *cagA*-negative strains were associated with a low bacterial load (110/119, 92.4%), negative *H. pylori* histology (82/90, 91.1%) and negative culture findings (119/131, 90%).

## 4. Discussion

Reliable and accurate diagnostic methods are mandatory for the effective management of gastroduodenal diseases associated with *H. pylori* infection. PCR-based diagnosis targeting at least two conserved genes can be considered the gold standard [15]. We evaluated the performance of both histology and culture for detecting *H. pylori* infection, taking as the gold standard molecular techniques (23S rRNA confirmed by *glmM)*.

The sensitivity of *Helicobacter*-like organisms histology detection by Giemsa staining was lower (73.3%) than in previous reports [21] but increased up to 94.2% in biopsies with high bacterial load (qPCR Ct < 30). Histology performance depends on the pathologist’s expertise, stain methods, prior antibiotics or PPI use and variable bacterial density according to the sampling site [21,22,23]. A real gap may exist between international guidelines and daily practice, with continued use of PPIs at endoscopy or one-region biopsy sampling, which can increase the likelihood of missing active infection by histology [24]. In line with a previous study in a pediatric population, in our study, the severity of gastritis was directly related to the presence of *Helicobacter*-like organisms on histology and high bacterial load by qPCR (Ct < 30) (Table 3) [25]. We confirmed that a false negative diagnosis by histology staining may be linked to biopsies with few *Helicobacter*-like organisms [26], in agreement with the findings of Benoit et al., stating that *H. pylori* is present only in cases of active gastritis and is always on the standard staining with H-E (in 94% of the cases) [27]. The authors concluded that it is not necessary to systematically perform a complementary histo- or immuno-histochemical technique on all gastric biopsies [27]. In cases of low bacterial load, *H. pylori* infection can be missed if histology is the only test performed.

*H. pylori* culture from gastric biopsy specimens has high specificity, but its sensitivity shows significant variations [28,29]. With our two-target PCR as a reference, culture showed perfect specificity (100%) and suboptimal sensitivity (74.4%). A previous study demonstrated increased sensitivity up to 90% if culture is performed under optimal conditions (i.e., fastidious growth requirements, rapid transportation of gastric biopsies to the laboratory) [30]. A false negative culture could be highly associated with poor bacterial load detected by qPCR [25]. Our findings of low sensitivity of histology and culture for specimens with low bacterial load are supported by a few studies of *H. pylori* gastric atrophy [31,32]. Indeed, density of *H. pylori* in the stomach mucosa decreases and may disappear completely during the late stages of atrophy, which is associated with markedly lower sensitivity of histology, culture and urease tests.

Molecular testing applied directly to biopsy specimens provides a fast and accurate diagnosis. One of the advantages is the assessment of all strains present in the specimen because some may grow on culture medium better than others [33]. This approach offers the possibility to evaluate bacterial quantification and detect antimicrobial resistance (mainly clarithromycin) and virulence factors.

In our adults, the *cagA* gene prevalence was 34.1%, in agreement with previous European findings, ranging from 30% to 60% [8], but lower than in other French findings, from 53% to 63% [34,35]. We found significantly lower *cagA* positivity in children than adults (*p* < 0.001), as previously observed [36]. By using immunoblotting, Rocha et al. found the detection of antibodies to the CagA antigen significantly higher in older children and hypothesized that increased *cagA* prevalence by age in children may reflect an evolutionary modification to achieve successful colonization during transmission [37]. The EPIYA-ABC motif was the most common in adults (80.5% of strains) as in children (85.7%) in our study, in line with prior reports from Europe [36]. Conversely, in East Asian countries, a high frequency of *H. pylori* strains with the more virulent EPIYA-D motif has been described in symptomatic children [38].

In our study, *cagA* positivity was significantly associated with increased severity of histology lesions, with a prevalence increasing from 7.3% in patients with normal findings, 27.1% in those with mild/moderate gastritis and 46.1% in those with severe gastritis (*p* < 0.001). These findings confirm that the histological virulence score is significantly increased in the presence of *cagA*-positive strains [39,40,41].

We found all combinations of the *vacA* s and m alleles. The distribution of *vacA* genotypes was similar to other findings from western Europe, with *s1m1* the most prevalent (51.2%), followed by *s2m2* (31.7%), *s1m2* (12.1%) and *s2m1* (5.0%) [42]. The *vacA* *s1m1* genotype has been associated with severe gastric lesions and increased risk of gastric cancer [43,44]. Our study showed that the more severe the histological lesions, the more prevalent the *vacA s1m1* allelic combination. However, this association was not significant because of the small number of patients with histology results and *vacA* genotyping. As previously reported, we confirmed the non-random distribution of genotype combinations of *cagA* and *vacA*: *cagA*-positive strains were frequently *vacA* *s1mA* and *cagA*-negative strains were frequently *vacA s2m2* (*p* < 0.001) [45].

This study is among the first attempts to assess the effects of virulence factors and bacterial load on the performance of diagnostic tests for *H. pylori* infection. Infection with a *cagA*-positive strain was associated with increased bacterial load (94.9%), which resulted in more frequent positive culture (94.3%) and *Helicobacter*-like organisms histology detection (91.7%). Conversely, *cagA* detection in strains was negative in 92.4% of biopsies with low bacterial load, 91.1% with negative *H. pylori* histology detection and 90% with negative culture. Some previous studies reported a relation between bacterial load in gastric biopsies or stools and the *cagA* virulence factor [10,46,47]. In 1996, Atherton et al. postulated that the *H. pylori* genotype may determine bacterial density, which in turn can determine inflammation level and epithelial injury [48]. Then, Belda et al. suggested that colonization by low virulence strains with slow multiplication in the gastric mucosa may not cause serious epithelial damage [46]. These differences can be linked to the bacterial density, which is itself linked to the presence/absence of CagA. These data may have an impact on the treatment by differentiating infection from colonization.

These hypotheses led to provocative questions. What is the clinical significance of the molecular detection of a low density of *cagA*-negative *H. pylori* usually missed by histology and conventional culture? Should we consider treating these cases with the same guidelines? Some studies tried to address this latter issue and showed that *cagA*-negative strains are a potential risk factor for treatment failure and may need a more thorough eradication protocol [49]. Nonetheless, the present study demonstrates that non-optimal sensitivity of culture and *H. pylori* histology determination can also be explained by a low density of *cagA*-negative *H. pylori* strains and not just poor pre-analytical conditions.

## 5. Conclusions

This study highlights the low sensitivity of conventional techniques (i.e., culture and *H. pylori* histology determination) leading to a false negative diagnosis of *H. pylori* infection if performed alone. Molecular-based techniques, using two different primers, provide an accurate detection of *H. pylori* and can be used to assess bacterial load and the virulence potential. *H. pylori* quantification associated with *cagA* genotyping in routine practice is essential to determine a sensitive and reliable diagnosis, to distinguish an infection from a colonization and to identify high-risk patients allowing management of eradication therapies.

## Figures and Tables

**Table 1:** Primer sequences used in this study.

Gene	Target Site	Primer	Primer Sequences (5′–3′)	PCR Product Size (pb)	References
23S rRNA	Domaine V	HPY-S	*AGGTTAAGAGGATGCGTCAGTC*	267	[12]
		HPY-AS	*CGCATGATATTCCCATTAGCAGT*		
*glmM*	*glmM*	glmM S	*GGATAAGCTTTTAGGGGTGTTAGGGG*	294	[14]
		glmM AS	*GCTTACTTTCTAACACTAACGCGC*		
*cagA*	*cagA* constant region	cagA-F	*GATAACAGGCAAGCTTTTGAGG*	349	[16]
		cagA-R	*CTGCAAAAGATTGTTTGGCAGA*		
	Empty-site	2	*ACATTTTGGCTAAATAAACGCTG*	360	[17]
		25	*TCATGCGAGCGGCGATGTG*		
	Forward for all EPIYA motifs	cagA28F	*TTCTCAAAGGAGCAATTGGC*		[18]
	EPYIA-A	cagA-P1C	*GTCCTGCTTTCTTTTTATTAACTTKAGC*	264	
	EPYIA-B	cagA-P2CG	*TTTAGCAACTTGAGCGTAAATGGG*	309	
	EPIYA-B	cagA-P2TA	*TTTAGCAACTTGAGTATAAATGGG*	309	
	EPYIA-C et D	cagA-P3E	*ATCAATTGTAGCGTAAATGGG*	468	
	EPIYA-D	cagA-pD	*TTGATTTGCCTCATCAAAATC*	486	[19]
	*cagA* variable region	cagA2530S	*GTTAARAATRGTGTRAAYGG* (*R = A* ou *G* and *Y = T* ou *C*)	Variable	[20]
		cagA3000AS	*TTTAGCTTCTGATACCGC*		
*vacA*	«s» region	VA1F	*ATGGAAATACAACAAACACAC*	259 (s1) 286 (s2)	[16]
		VA1R	*CTGCTTGAATGCGCCAAAC*		
	«m» region	VAGF	*CAATCTGTCCAATCAAGCGAG*	570 (m1) 645 (m2)	[16]
		VAGR	*GCGTCTAAATAATTCCAAGG*		

**Table 2:** Association of bacterial load by qPCR with biopsy culture results (*n* = 513) and *Helicobacter pylori* histology detection (*n* = 338, 278 adults and 60 children).

	Bacterial Load (qPCR Ct Values)		*p*-Value
	High (Ct < 30) *n* (%)	Low (Ct ≥ 30) *n* (%)	
Culture (*n* = 513) *			
Positive	368 (93.4)	14 (11.8)	<0.001 ^Φ^
Negative	26 (6.6)	105 (88.2)	
Total	394 (100)	119 (100)	
*H. pylori* histology detection (*n* = 338) **			
Positive	234 (92.5)	14 (16.4)	<0.001 ^Φ^
Negative	19 (7.7)	71 (83.5)	
Total	253 (100)	85 (100)	

^Φ^ chi-square test; Ct = cycle threshold; * Culture was evaluated in 513 *H. pylori*-positive biopsies by comparison to the gold standard considered in this study (23S rRNA qPCR confirmed by *glmM* amplification). ** *H. pylori* histological detection was evaluated in 338 *H. pylori*-positive biopsies for which histology reports were available.

**Table 3:** Association between presence of *H. pylori* on histology, bacterial load (qPCR), *cagA* status of the strain and severity of histology lesions in 338 *H. pylori*-positive biopsies (278 adults and 60 children).

	Histology Grading			*p*-Value
	Normal *n* (%)	Mild and Moderate Gastritis *n* (%)	Severe Gastritis/Gastric Cancer *n* (%)	
Age group				
Adults (*n* = 278)	23 (56.1)	204 (87.9)	51 (78.5)	-
Children (*n* = 60)	18 (43.9)	28 (12.1)	14 (21.5)	
Total	41 (100)	232 (100)	65 (100)	
*H. pylori* histology detection				
Positive	3 (7.3)	191 (82.3)	54 (83)	<0.001 ^Φ^
Negative	38 (92.7)	41 (17.7)	11 (16.9)	
Total	41 (100)	232 (100)	65 (100)	
Bacterial load (qPCR Ct values)				
High (Ct < 30)	5 (12.2)	193 (83.2)	56 (86.1)	<0.001 ^Φ^
Low (Ct ≥ 30)	36 (87.8)	39 (16.8)	9 (13.8)	
Total	41 (100)	232 (100)	65 (100)	
*cagA* status				
Positive	3 (7.3)	63 (27.1)	30 (46.1)	<0.001 ^Φ^
Negative	38 (92.7)	159 (68.5)	34 (52.3)	
Undetermined	0 (0)	10 (4.3)	1 (1.5)	
Total	41 (100)	232 (100)	65 (100)	

^Φ^ chi-square test; Ct = cycle threshold.

**Table 4:** Association between culture positivity, bacterial load (qPCR Ct values), presence of *H. pylori* on histology and *cagA* status of the strain.

	*cagA* Status			*p*-Value
	Positive *n* (%)	Negative *n* (%)	Undetermined *n* (%)	
Culture (*n* = 513) *				
Positive	149 (94.3)	218 (64.7)	15 (83.3)	<0.001 ^Φ^
Negative	9 (5.7)	119 (35.3)	3 (16.7)	
Total	158 (100)	337 (100)	18 (100)	
Bacterial load (*n* = 513) *				
High (Ct < 30)	150 (94.9)	227 (67.3)	17 (94.4)	<0.001 ^Φ^
Low (Ct ≥ 30)	8 (5.1)	110 (32.7)	1 (5.6)	
Total	158 (100)	337 (100)	18 (100)	
*H. pylori* histology detection in 338 available histological reports **				
Positive	88 (91.7)	149 (64.5)	11 (100)	<0.001 ^Φ^
Negative	8 (8.3)	82 (35.5)	0 (0)	
Total	96 (100)	231 (100)	11 (100)	

^Φ^ chi-square test; Ct = cycle threshold; * Culture and bacterial load were evaluated in 513 *H. pylori*-positive biopsies by comparison to the gold standard considered in this study (23S rRNA qPCR confirmed by *glmM* amplification). ** *H. pylori* histology detection was evaluated in 338 *H. pylori*-positive biopsies for which histology reports were available.
